# Pattern-Based Decoding for Wi-Fi Backscatter Communication of Passive Sensors

**DOI:** 10.3390/s19051157

**Published:** 2019-03-07

**Authors:** Hwanwoong Hwang, Jae-Han Lim, Ji-Hoon Yun, Byung Jang Jeong

**Affiliations:** 1 Department of Electrical and Information Engineering, Seoul National University of Science and Technology, Seoul 01811, Korea; hwanwoong@seoultech.ac.kr; 2 Department of Software, Kwangwoon University, Seoul 01897, Korea; 3 Electronics and Telecommunications Research Institute, Daejeon 34129, Korea; bjjeong@etri.re.kr

**Keywords:** ambient backscatter communication, sensor network, ultralow power communication, sensor tag, IoT

## Abstract

Ambient backscatter communication enables passive sensors to convey sensing data on ambient RF signals in the air at ultralow power consumption. To extract data bits from such signals, threshold-based decoding has generally been considered, but suffers against Wi-Fi signals due to severe fluctuation of OFDM signals. In this paper, we propose a pattern-matching-based decoding algorithm for Wi-Fi backscatter communications. The key idea is the identification of unique patterns of signal samples that arise from the inevitable smoothing of Wi-Fi signals to filter out noisy fluctuation. We provide the mathematical basis of obtaining the pattern of smoothed signal samples as the slope of a line expressed in a closed-form equation. Then, the new decoding algorithm was designed to identify the pattern of received signal samples as a slope rather than classifying their amplitude levels. Thus, it is more robust against signal fluctuation and does not need tricky threshold configuration. Moreover, for even higher reliability, the pattern was identified for a pair of adjacent bits, and the algorithm decodes a bit pair at a time rather than a single bit. We demonstrate via testbed experiments that the proposed algorithm significantly outperforms conventional threshold-based decoding variants in terms of bit error rate for various distances and data rates.

## 1. Introduction

Ambient backscatter communication is widely considered a means of ultralow power communication of low-end passive sensors (e.g., sensor tag) in Internet of Things (IoT) environments. Ambient backscatter communication is realized by letting a sensor tag reflect and absorb ambient signals in the air according to sensing-data bits to transmit by controlling the state of a radio frequency (RF) switch. For example, a tag reflects ambient signals (reflection state) for transmitting data one, but absorbs ambient signals (absorption state) for transmitting data zero; a receiver then sees the amplitude changes of received signals from which it can decode data bits. A variety of ambient signals, such as TV broadcasts, Wi-Fi and FM radio, are considered for this purpose. In particular, Wi-Fi backscatter communication is promising since Wi-Fi access points (main signal sources) prevail and most smartphones/tablet PCs (data-collecting node or Internet gateway for Wi-Fi backscatter tags) are already equipped with a Wi-Fi transceiver. Such wide availability of Wi-Fi-equipped devices is the key advantage of Wi-Fi backscatter communication for the quick deployment of technology. Thus, Wi-Fi has been considered as a promising carrier source of backscatter communication in the literature [1,2,3,4,5,6,7].

Despite the advantage of Wi-Fi backscatter communication, decoding data bits from backscattered Wi-Fi signals is challenging since a Wi-Fi signal itself has inherent fluctuations due to the high peak-to-average-power-ratio (PAPR) nature of orthogonal frequency-division multiplexing (OFDM). Prior studies [1,8,9,10] employed a naive approach for decoding, whereby the amplitudes of signal samples are compared with a threshold to be classified between data one and zero. The downside of this approach is that a receiver can reliably decode data bits only if ambient signals are typically more stable (less fluctuating) than Wi-Fi signals. In addition, decoding reliability would significantly be reduced if we allow faster switching between reflection and absorption states for a higher data rate (e.g., over a few hundred kbps). Moreover, the threshold that has been determined based on past signal samples may not always be applicable to forthcoming samples due to changing fluctuation patterns of Wi-Fi signals and wireless channels.

In this paper, we propose a simple but novel algorithm to decode data bits from backscattered Wi-Fi signals. The key idea is the identification of unique patterns of signal samples that arise from the inevitable smoothing of Wi-Fi signals to filter out noisy fluctuation. We prove that the pattern of smoothed signal samples is obtained as the slope of a line using a closed-form equation. On this mathematical basis, we designed the proposed algorithm to identify the pattern of the changes of received signal samples as a slope, rather than classifying their amplitude levels. Hence, our algorithm is more robust to signal fluctuation than the previous threshold-based decoding variants and does not need tricky threshold configuration. To further improve reliability, the pattern is identified for a pair of adjacent bits, and the algorithm decodes a bit pair at a time rather than a single bit. This behavior enables the algorithm to make a decision for a bit from two patterns (one from a pair with the previous bit and the other from a pair with the next bit), thus making the decision more reliable against occasional fluctuation peaks.

We demonstrate via testbed experiments that the proposed algorithm outperforms conventional threshold-based decoding variants in terms of a bit error rate (BER), reducing it by up to 94%.

The prototype is based on the simplest design of a backscatter tag that data bits are modulated on Wi-Fi signals by binary switching, like Reference [1]. Since the proposed algorithm aims for reliable decoding from observation of signal changes, it can also be combined with or extended for other techniques, e.g. frequency shift for a longer communication range [7,11], high-order modulation like quadrature amplitude modulation (QAM) [12,13].

The rest of the paper is organized as follows. In Section 2, we review related work. Section 4 describes the system model. Section 3 then presents important observations on the patterns of a smoothed Wi-Fi signal. The proposed algorithm is described in Section 5. The experimental setup and performance results are shown in Section 6. Finally, Section 7 concludes the paper.

## 2. Related Work

There have been many research works on backscatter communications with various types of ambient signals. Liu et al. [8] introduced ambient backscatter communication with TV signals that are available nationwide, thus not needing any infrastructure deployment. In Reference [9], two antennas are used by a backscatter receiver to reduce the direct-link interference of TV signals. In Reference [10], the authors used a full-duplex technique to enable immediate feedback by a tag on the same frequency channel. Wang et al. [14] designed backscatter communication for FM radio signals. EkhoNet [15] implemented backscatter communication on UMass Moo and WISP platforms. In Reference [16], the authors proposed a universal backscatter reader that can receive backscattered signals for all available ambient broadcast radio sources. LoRea [17] is a backscatter architecture that combines techniques such as narrow-band backscatter transmissions and self-interference mitigation to achieve long communication range. Braidio [18] dynamically switches between active and passive communication modes according to the available energy. Some research works proposed tag designs for high-order QAM modulation [12,13]. For reliable decoding of high-order modulated signals against noisy Wi-Fi carrier signals, the approach of the proposed algorithm can still be used, thus enabling better detection of amplitude changes as well as phase changes.

Recent work studied Wi-Fi backscatter communication. In Reference [1], a tag backscatters transmissions from a Wi-Fi router, and off-the-shelf Wi-Fi devices decode data bits using per-frame CSI/RSSI values. BackFi [2] improves the data rate of Wi-Fi backscatter communication with a tag’s phase-changing modulation and a full-duplex radio at the Wi-Fi router, which is a signal source and a receiver at the same time, to decode backscattered signals against self-interference, but at the expense of an increase of complexity. Passive Wi-Fi [3] generates 802.11b-compatible signals to transmit data bits by backscattering other Wi-Fi signals so that any Wi-Fi device can decode the signals. The work was extended in Reference [4] to generate 802.11b signals from the signals of Bluetooth devices via backscattering. Yang and Liang proposed a transceiver design for backscatter communication to cancel out direct-link interference over an OFDM carrier in Reference [5], and considered multiantenna receivers in Reference [6]. HitchHike [7] performs codeword translation of a received Wi-Fi signal at a tag and generates a signal decodable by a standard 802.11b receiver. FreeRider [19] extends HitchHike for communication with multiple commodity radios such as 802.11g/n Wi-Fi, ZigBee and Bluetooth with the support of multiple tags. Our algorithm requires more information (I/Q samples) from a Wi-Fi receiver chip, but does not rely on a specific version of 802.11. Frequency shift adopted by HitchHike [7] and FS-Backscatter [11] greatly enhances the communication range of Wi-Fi backscatter communication by avoiding the strong interference of a Wi-Fi carrier signal at the receiver side. Our algorithm can also be combined with frequency shift to improve its communication range.

There have also been theoretical research works. Kang et al. [20] considered two antennas at a backscatter transmitter and an energy detector that uses the difference in energy levels of the received signals to detect bits at a backscatter receiver. Yang et al. [21] introduced a successive interference cancellation (SIC)-based detector that can achieve near-ML detection performance. Wang et al. [22] formulated a transmission model, designed a data-detection algorithm, and derived a detection threshold in a closed form. In Reference [23], BER performance was derived when 8-PSK modulation and a noncoherent detector were adopted. In Reference [24], three-state switching between reflecting, nonreflecting, and negative-reflecting states was proposed; in the negative-reflecting state, a tag reflects RF signals in an inverse phase. Theoretical modeling of backscatter-communication networks based on stochastic geometry was conducted in References [25,26]. In Reference [27], the energy-saving of backscatter communication was formulated as an optimization problem in delivering data with energy-consumption disparity. Darsena et al. [28] investigated the performance limits of ambient backscatter communication systems employing OFDM, in terms of information-theoretic metrics, such as the ergodic and outage capacity. While the proposed algorithm is dedicated to reliable decoding, integrating it with other techniques into a system may help for getting closer to the achievable capacity of the reference.

Applications of Wi-Fi backscatter other than data communication were considered in some research work. A handwriting input system called Word-Fi was introduced in Reference [29], which mitigates surrounding noise and extracts the weak signals incurred by tiny writing gestures. WiTag [30] finds the location of a Wi-Fi backscatter tag by extracting information such as amplitude, phase, and frequency changes that are associated with the tag’s location from the received signals.

## 3. System Model

**Figure 1:**
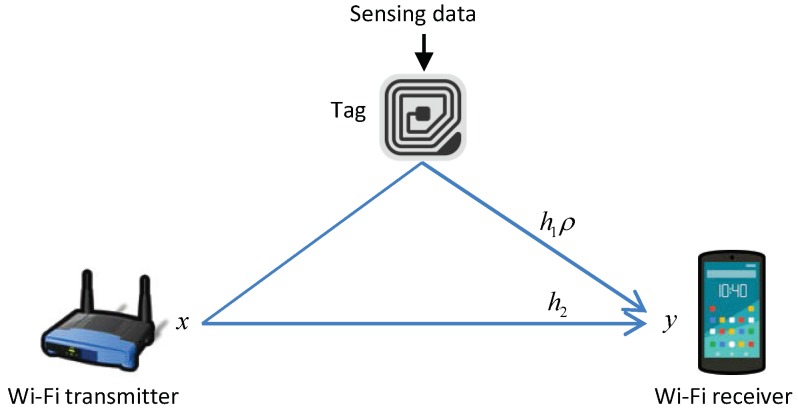
System model of Wi-Fi backscatter communication.

The communication system under consideration is depicted in Figure 1. A background signal, i.e., Wi-Fi frame, is transmitted from a Wi-Fi transmitter (e.g., AP) to a Wi-Fi receiver (e.g., smartphone). A backscatter tag modulates its bits on received Wi-Fi signals; the bits to transmit are either coded or uncoded, and each bit value (one or zero) is mapped to an RF switch’s state (reflection or absorption). The backscattered signal is then received by the Wi-Fi receiver as the sum of the signals along both paths. The received signal is expressed as

(1) $$y\left[k\right]=h_{1}\rho r\left[k\right]x\left[k\right]+h_{2}x\left[k\right]+\sigma \left[k\right]$$

where *x* and *y* are the amplitudes of the transmitted and received Wi-Fi signal samples, respectively, $h_{1}$ and $h_{2}$ are the channel gains of backscattered and other signal components, respectively (for simplicity, they are assumed static during a single frame period), ρ is the reflection loss of the tag, r∈{1,0} is the switching state of the tag (1 is reflection and 0 is absorption), and σ is the noise floor including thermal noise; *k* is the sample index.

We assume that the backscatter tag reflects signal (r=1) for data one, and this results in an increased (high) amplitude level of the backscattered signal; it absorbs the signal (r=0) for data zero, and the backscattered signal has no increased (low) amplitude level. The tag maintains its switching state for period *T* (given as the number of signal samples), which we call a slot (one bit is transmitted during a slot) and can switch the state at the end of each slot depending on the value of the next bit to transmit. Slot *n* is represented as $\left[\tau_{n},\tau_{n}+T\right]$ (samples), where $\tau_{n}$ is the first sample of slot *n*.

We assume that the bits to transmit are coded by, but not limited to, FM0 coding [8], i.e., the code rate is 1/2 (one information bit needs the transmission of two coded bits). In FM0 coding, if the information bit to transmit is data one, the signal level is maintained (represented by bit pair (1, 1) or (0, 0)); if it is data zero, the level is inverted in the middle (represented by bit pair (0, 1) or (1, 0)). The signal level is inverted at the end of every slot. Unless otherwise specified, the transmission bit rate is represented as the transmitted number of coded bits per unit time.

We limited the focus of the paper to the communication scenario where a backscatter tag transmits its data to more powerful receiver nodes, such as smartphones and IoT gateways, which is common in IoT environments, in that things are equipped with sensors and report sensing results to the outer world (e.g., the Cloud).

In addition, we make the following assumptions.

**Assumption** **1.** *Digital-domain processing of received signal samples: Receiver nodes are assumed to have sufficient computing power and thus run a decoding process in the digital domain for better performance.*

**Assumption** **2.** *In-frame switching: To achieve a higher transmission bit rate with occasional Wi-Fi signals, a backscatter device changes its switching state within a Wi-Fi frame reception so that it modulates multiple data bits on the frame.*

## 4. Patterns in Smoothed Wi-Fi Signal

**Figure 2:**
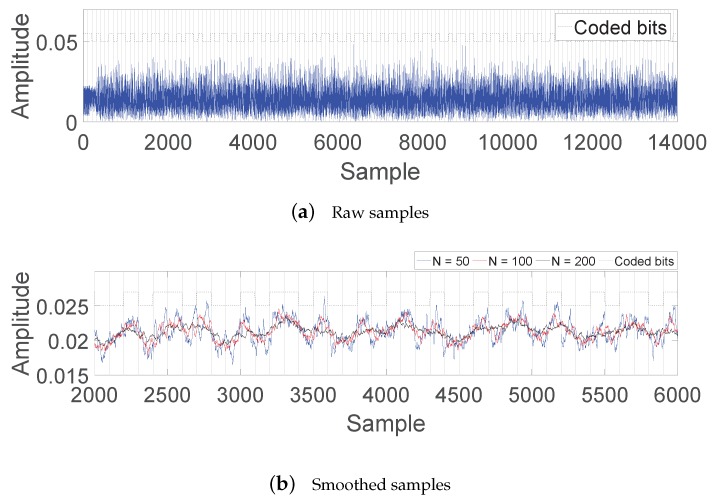
Samples of a received Wi-Fi frame with backscattering for variously sized average windows.

Figure 2a shows the received signal samples of a backscattered Wi-Fi data frame for which a backscatter tag modulates coded bits at 100 kbps. The beginning non-OFDM part of the frame (32 μs for a preamble and part of the physical-layer header) has small fluctuation, but the following OFDM-modulated part shows considerable fluctuation. Due to such fluctuation of the Wi-Fi signal itself, no amplitude patterns of the tag’s backscattering are noticeable in raw signal samples.

In general, a backscatter tag’s bit modulation on a Wi-Fi signal is at a much lower rate than for Wi-Fi. Therefore, we can smooth the fluctuation of the Wi-Fi signal out while letting backscattered amplitude patterns remain [8]. To this end, we applied moving average to signal samples, i.e., a window of signal samples were averaged to produce another signal value, which is a representative low-complexity method of low-pass filtering. The moving average process we considered is given as

(2) $$\bar{y}\left[i\right]=\frac{1}{N}\sum_{k=i-N/2+1}^{i+N/2}y\left[k\right]$$

where *y* and $\bar{y}$ are the amplitudes of received Wi-Fi signal samples before and after the moving average, respectively, and *N* is the window size (in the number of samples) of the moving average.

The signal samples after smoothing are shown in Figure 2b with several values of window size *N*. It was observed that a significant amount of fluctuation disappeared, and amplitude patterns were then noticeable. However, the following points show that conventional threshold-based decoding still suffers:

- As the window size of the moving average increases, the fluctuation of the Wi-Fi signal is better reduced, but backscattered amplitude patterns are also smoothed out more, which becomes more severe for a higher backscatter bit rate. That is, the window size of the moving average is one of the factors determining decoding performance.
- The entire samples of a slot period are not above or below a threshold. This results not only from residual fluctuation, but also from the nature of the moving average. Thus, a small variation of the signal may lead to the wrong decision of threshold-based decoding for an information bit.
- The scale of amplitude changes is significantly reduced. Therefore, a small variation of a threshold from the optimal one may result in decoding failures.
- The scale of amplitude changes changes depending on the window size of smoothing and the switching rate of a backscatter tag. This means that an adequate threshold has to be adaptive to such parameters, which makes threshold configuration even more difficult.

**Figure 3:**
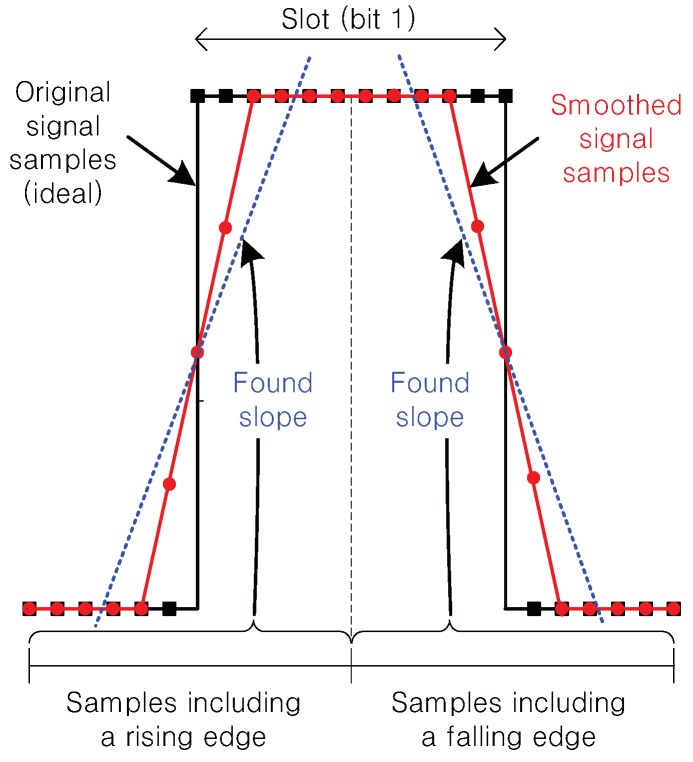
Illustration of signal-smoothing via moving average, and slope finding via first-order curve-fitting for ideal (fluctuation-free) signal samples.

To tackle the above challenges, we paid attention not to amplitude levels, but to the pattern of its changes after signal smoothing was applied. Ideally, as illustrated in Figure 3, if a tag alternates reflection and absorption states, the signal is modulated as a square wave. When the signal goes through the smoothing (moving average) of Equation (2), the distortion patterns are twofold: (1) inclining slope around a rising edge; (2) declining slope around a falling edge. Suppose that the tag changes the switching state at 1/THz, i.e., the reflection state remains unchanged during *T* (in the number of samples). In order not to filter out the reflection patterns of the tag, we must let N<T. Then, these signal-distortion patterns after smoothing are formally proved in the following proposition.

**Proposition** **1.** *If N is large enough to filter out the signal fluctuation resulting from OFDM, channel-gain variations, and thermal noise, while N≤T, $\bar{y}$ of Equation (2) monotonically increases (decreases) when the window of signal samples for smoothing includes a rising (falling) edge.*

**Proof.**  Under the above assumption of *N*, applying Equation (2) to Equation (1) leads to

(3) $$\bar{y}\left[i\right]=\frac{h_{1}\rho \bar{x}}{N}\sum_{k=i-N/2+1}^{i+N/2}r\left[k\right]+h_{2}\bar{x}+\bar{\sigma }$$

where $\bar{x}$ is the average of *x* samples within the window. When the window of signal samples for smoothing includes a rising edge at *j*, r[k]=1 for k≥j, and otherwise zero. Then, the last term of the above equation becomes $\frac{h_{1}\rho \bar{x}}{N}\left(i+N/2-j\right)$, thus making $\bar{y}\left[i\right]$ monotonically increase for *i*. When *N* includes a falling edge at *j*, the last term becomes $\frac{h_{1}\rho \bar{x}}{N}\left(j-i+N/2-1\right)$, and now $\bar{y}\left[i\right]$ monotonically decreases for *i*. □

This observation motivates us to exploit such patterns for decoding data bits. The following proposition shows that we can identify inclining and declining signal patterns as positive and negative gradients (slopes) of a line, respectively, via simple first-order curve fitting (linear regression) whose solution is given in a closed form.

**Proposition** **2.** *First-order least-mean-square curve fitting of any monotonically increasing (decreasing) function produces a line with a positive (negative) gradient.*

**Proof.**  The first-order least-mean-square curve-fitting problem is presented as minimizing the cost function of $\sum_{i}{\left(v_{i}-\left(a+bu_{i}\right)\right)}^{2}$, where $u_{i}$ and $v_{i}$ are input and output samples, respectively, and *a* and *b* are the coefficients of the curve (problem variables in curve fitting). By letting the cost’s partial derivatives of *a* and *b* be zero and solving the resulting simultaneous equations, we obtain the optimal *b* as [31]

(4) $$b^{*}=\frac{\sum_{i}u_{i}v_{i}-m\bar{u}\bar{v}}{\sum_{i}{\left(u_{i}-\bar{u}\right)}^{2}}=\frac{\mathrm{cov}(u,v)}{\sigma_{u}^{2}}$$

where $\bar{u}$ and $\bar{v}$ are the averages of *u* and *v*, respectively, for the given samples, $\sigma_{u}^{2}$ is the variance of *u*, *m* is the number of samples, and cov() is the covariance. It is known that cov(f(u),g(u))≥0 holds for any two increasing functions *f* and *g*, and if one is increasing and the other is decreasing, then the inequality is reversed. Therefore, if *v* is a monotonically increasing function of *u*, cov(u,v)≥0 and the optimal *b* is positive. Conversely, if *v* is a monotonically decreasing function of *u*, cov(u,v)<0, and the optimal *b* becomes negative [32,33]. □

**Proposition** **3.** *If N is large enough to filter out signal fluctuation while N≤T, the gradient of the fitted line for moving-averaged samples with N is always positive (negative) when the window of smoothing includes a rising (falling) edge.*

**Proof.**  The proof is straightforward according to Propositions 1 and 2; thus, it is omitted. □

**Figure 4:**
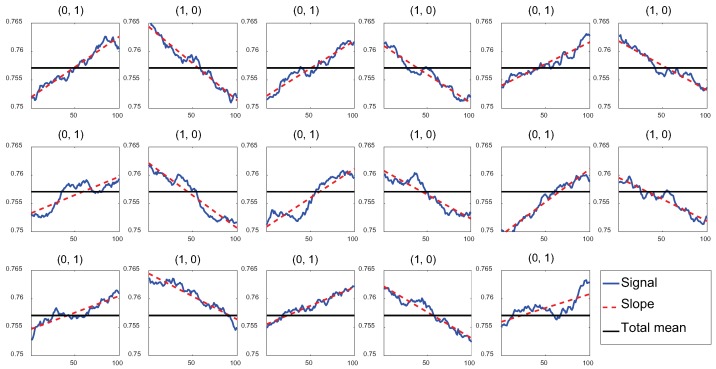
Signal samples after smoothing for different bit pairs and corresponding first-order curve-fitting results.

Figure 4 shows the groups of signal samples for a set of bit pairs that appear in the smoothed signal of Figure 2a; we chose those of bit pairs (0, 1) and (1, 0) for straightforward illustration. The horizontal line is the average amplitude of the whole Wi-Fi frame, which is the threshold of decoding configured by the total-mean method. In an ideal condition, the first half of samples are below the line and the second half are above it for bit pair (0, 1); the opposite trend is shown for bit pair (1, 0), i.e., the signal of each case crosses the horizontal line at the center. In such a case, we achieve a reliable decoding decision of the total-mean method. However, the real samples are not aligned with the expectation. Moreover, several cases show that most samples are below or above the line, thus leading to a decoding error. For example, the 12th group has most samples below the threshold, and the 14th group has most samples above the threshold. Despite such irregular signal fluctuation, signal patterns are still reserved in all cases. The line found by first-order curve fitting for each group of samples is shown as a red dotted line; rising-edge groups corresponding to bit pair (0, 1) produce positive-gradient lines (the signal inclines) and falling-edge groups corresponding to bit pair (1, 0) produce negative-gradient lines (the signal declines).

## 5. Pattern-Based Decoding Algorithm

Based on the observations made in the previous section, we developed a pattern-based decoding algorithm. The pseudocode of the algorithm is given in Algorithm 1.
**Algorithm 1** Pattern-based decoding algorithm.1:**for all** slot *n* **do**2: slope[*n*] = GetSlope($\left[\tau_{n}-T/2,\tau_{n}+T/2-1\right]$)3: **if** slope[*n*] > 0 **then**4:  bitPair[*n*] = (0, 1)5: **else if** slope[*n*] < 0 **then**6:  bitPair[*n*] = (1, 0)7: **end if**8: bit[*n*] = bitPair[*n*][2]9: **if**|slope[*n*]|≥|slope[n−1]|**then**10:  bit[n−1] = bitPair[*n*][1]11: **end if**12:**end for**

In order to decode the bit of slot *n*, we designed the algorithm to identify the pattern of samples in $\left[\tau_{n}-T/2,\tau_{n}+T/2-1\right]$, i.e., T/2 samples from slot n−1 and the other T/2 samples from slot *n*, not in $\left[\tau_{n},\tau_{n}+T-1\right]$, due to the following reasons ($\tau_{n}$ is the first sample of slot *n*):

- When signal smoothing of Equation (2) is applied, the sample pattern in $\left[\tau_{n}-T/2,\tau_{n}+T/2-1\right]$ monotonically increases and decreases if the transmitted signal at $\tau_{n}$ corresponds to rising and falling edges, respectively, according to Proposition 1, thus being identified as positive and negative slopes, respectively, according to Proposition 3.
- It does not identify a single bit value, but the transition of two adjacent bits at once. This means that we decode the value of a bit twice, once with the previous bit and the second time with the next bit. Therefore, decoding becomes more robust against sudden unstable noisy samples.

The algorithm calculates slope of samples $\left[\tau_{n}-T/2,\tau_{n}+T/2-1\right]$ (denoted by slope[*n*]) via linear curve fitting, which is derived from Equation (4) as (the details of the derivation are given in Appendix A):

(5) $$\mathrm{slope}\left[n\right]=\frac{12}{T(T^{2}-1)}\sum_{i=\tau_{n}-T/2}^{\tau_{n}+T/2-1}\left(i-\tau_{n}+\frac{1}{2}\right)\bar{y}\left[i\right]$$

If the slope is positive (inclining pattern), the algorithm assumes that the samples correspond to bit pair (0, 1); otherwise, to bit pair (1, 0). Since the first T/2 samples of pattern detection are from the previous slot n−1, the algorithm identifies the second value of the resulting bit pair (bitPair[*n*][2]) as the bit of slot *n* (bit[*n*]). Finally, the algorithm compares slope[*n*] obtained for slot *n* with that for the previous slot (slope[n−1]). If slope[*n*] is larger than the previous one, the algorithm assumes that the detected bit pair from slope[*n*] (bitPair[*n*]) is more confident and modifies the bit value of the previous slot (bit[n−1]) as the first value of this bit pair (bitPair[*n*][1]).

In the algorithm, for both bit pairs (1, 1) and (0, 0), the slope is ideally obtained as zero. Then, the corresponding bit value is judged from the samples of the next slot because the samples of the next slot are either (1, 0) or (0, 1), having a nonzero slope (no consecutive bit pairs of either (1, 1) or (0, 0) appear in FM0 coding). In reality, for both bit pairs (1, 1) and (0, 0), the slope may be obtained as a nonzero value, but its absolute value is probably smaller than the one for the next slot. Therefore, from the comparison between the slopes for two consecutive slots, the algorithm is likely to pick the bit value judged from the samples for the next slot.

**Figure 5:**
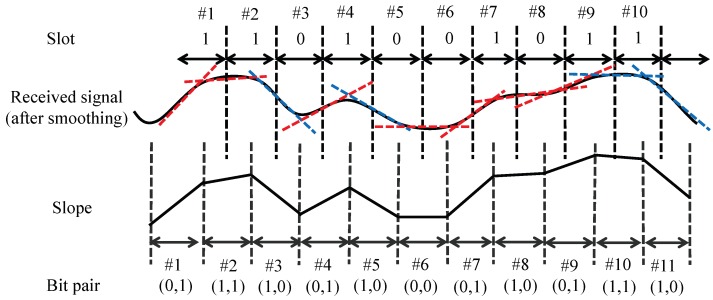
Illustration of the pattern-based decoding algorithm.

Figure 5 illustrates the operation of the algorithm. The transmitted bits are the FM0-coded ones for the data of alternating one and zero. Suppose that bit[1] is decoded as data one, and slope[1] is positive. In order to decode bit[2], the algorithm calculates slope[2], which is obtained small but positive, and judges bit[2] as data one. The previous slope[1] is for bit pair (0, 1) and larger than slope[2]; thus, the algorithm keeps the decision of bit[1]. The algorithm obtains slope[3] as a negative since it is for bit pair (1, 0), and judges bit[3] as data zero. The absolute value of slope[3] is larger than that of slope[2]; thus, bit[2] is determined as the first value of bit pair (1, 0), i.e., data one. The calculated slope for bit[4] corresponds to bit pair (0, 1) and is obtained as positive, thus resulting in bit[4] as data one. Since slope[3] and slope[4] produce bit pairs (1, 0) and (0, 1), respectively, a comparison between two does not change the result of bit[3]. The same procedure is applied to bit[5]–bit[7]. However, for bit[8], slope[8] is obtained positive, which is not aligned with the true bit pair (1, 0) due to signal fluctuation. The algorithm assumes that the corresponding bit pair is (0, 1) and judges bit[8] as data one, which is corrected by the decision of slope[9], since slope[9] is obtained larger than slope[8].

## 6. Experimental Evaluation

In this section, we evaluate the proposed pattern-based decoding algorithm through indoor testbed experiments. For comparison with conventional threshold-based decoding, we considered two methods to configure a threshold: (1) the average of all samples of a received Wi-Fi frame (total mean), and (2) the average of the samples of a sliding window (sliding). In the sliding method, window size was set as the same as a slot length.

### 6.1. Environmental Setup

**Figure 6:**
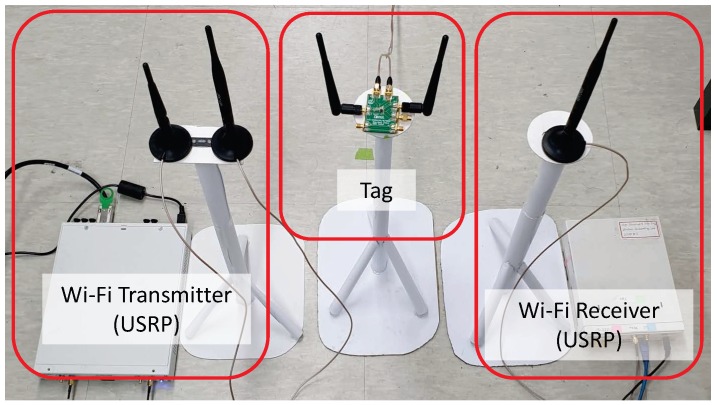
Experimental setup.

We used two Ettus Universal Software Radio Peripherals (USRPs, N210 and X310) [34] to generate and receive Wi-Fi signals, as shown in Figure 6. The gr-ieee802-11 module of GNU Radio [35] was used to generate Wi-Fi carrier signals according to the OFDM frame format of IEEE 802.11g. Each Wi-Fi frame is composed of a 64 μs physical-layer header and a following MAC frame of 1528 bytes. It is transmitted at a bit rate of 9 Mbps (QPSK modulation and 3/4 code rate) and 1.4 ms long. The USRPs operate at the center frequency of 2.432 GHz. The transmit power of the transmitting USRP is 14.78 dBm (as measured at the output antenna port by a network analyzer). The receiving USRP captures the I/Q signals of received Wi-Fi frames via two antennas, each at a sampling rate of 10 MHz (for moderate computational loads), and preprocesses them according to the μMO procedure [9], so that communication distance is expanded thanks to diversity. Then, the resulting signal stream is smoothed via moving average with the window size same as the slot length and input to the processing block of bit decoding.

The backscatter tag was implemented using Analog Devices’ RF switch ADG902 [36] for the reflection and absorption of ambient Wi-Fi signals. Information data were generated as alternating one and zero, and coded using FM0 coding [8]. Then, the backscatter tag transmits coded bits at a bit rate of 20, 50, 100, and 200 kbps. The total number of Wi-Fi frames generated for each experiment was 500, where each of received Wi-Fi frame is represented as 14,000 signal samples. The performance metric is the bit error rate (BER) of information bits for various tag distances and bit rates.

**Figure 7:**
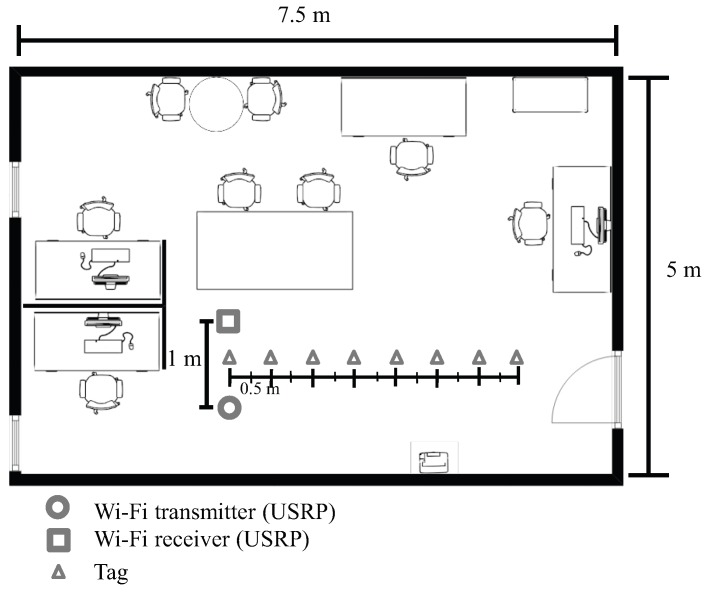
Experimental setup for evaluation with various tag distances.

**Figure 8:**
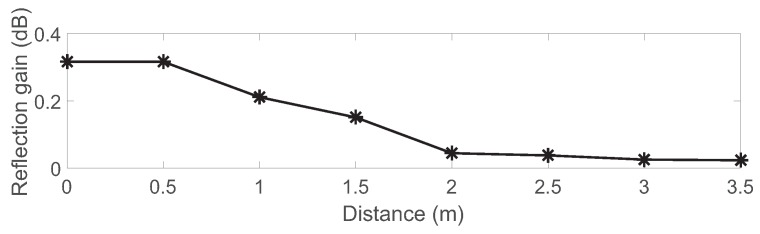
Tag reflection gain.

Figure 6 and Figure 7 show the experimental setup, and the placement of the USRPs and the backscatter tag in the indoor environment of a small office. The USRPs were one meter apart from each other. The backscatter tag was placed in the middle of the USRPs and moved to eight different positions, each 0.5 m apart from the adjacent ones. All were at a height of 0.5 m from the ground. The tag’s reflection gain (calculated as the ratio of the backscattered signal amplitude of the tag’s reflection state to that of the absorption state, i.e., approximately $|h_{1}\rho +h_{2}\left|/\right|h_{2}|$ from Equation (1)) for different distances is given in Figure 8.

### 6.2. Experimental Results

**Figure 9:**
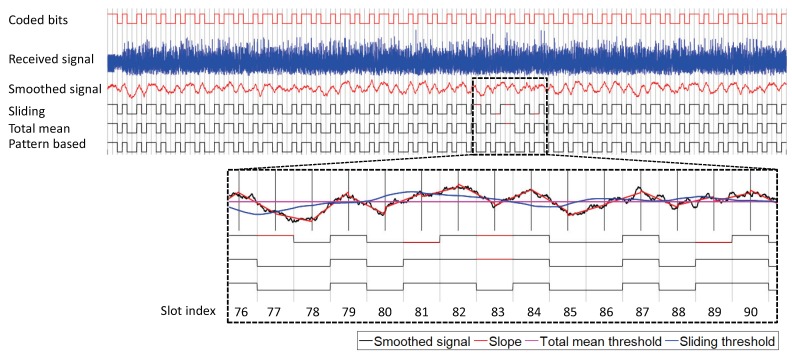
Illustration of the decoding result of different methods for an example Wi-Fi frame with backscattering at a bit rate of 100 kbps.

Figure 9 shows a signal trace of the experiment where the backscatter tag transmits the shown stream of bits at 100 kbps; each slot is 10 μs long and the Wi-Fi frame lasts 1.4 ms. Thus, the backscatter tag transmits up to 70 information bits (140 coded bits) on the frame. It also shows more details about where bit errors occur. In the raw samples of the received signal, it is hard to notice the amplitude patterns corresponding to the bit stream. After smoothing, the received signal now shows noticeable amplitude patterns. The bits with decoding error are shown as red in the figure; four bit errors occurred in the sliding method, one bit error occurred in the total mean method, and no bit error occurred in the pattern-based method. In slot 83, in which a bit error occurred for both threshold-based methods, the smoothed signal had a convex downward shape, but more samples were above both thresholds and demodulated as data one. On the other hand, the pattern-based method checks the two adjacent slopes. Since slope[83] is negative, the bit pair is judged as (1, 0), and data zero is the candidate for bit[83]. Since slope[84] is positive, the bit pair is detected as (0, 1); thus, both slopes confirm that bit[83] is data zero.

**Figure 10:**
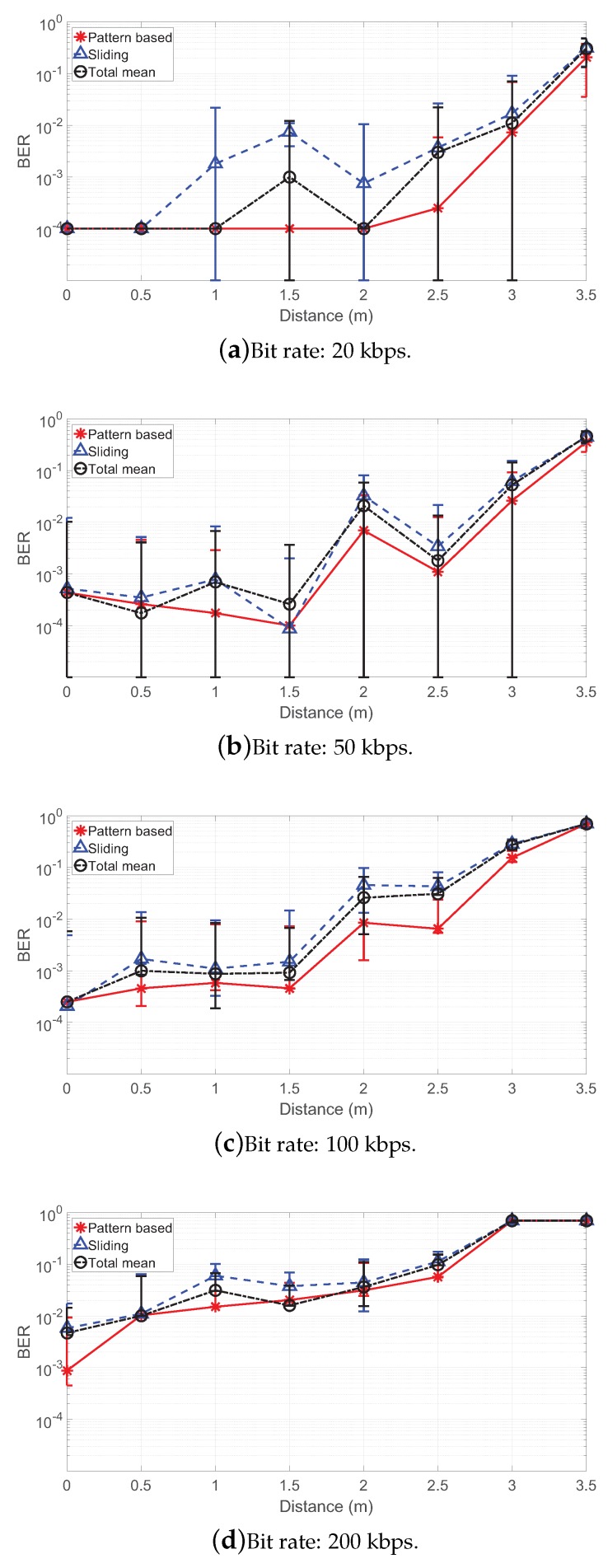
Bit error rate (BER) results for different tag positions.

Figure 10 shows the comparison of BER results between decoding algorithms for different tag distances. In the figures, it is clearly shown that the proposed decoding algorithm outperforms threshold-based ones in most positions for all bit-rate cases. In particular, the proposed algorithm achieves a BER of $2.5e^{-4}$, $1.1e^{-3}$, $6.5e^{-3}$ and $5.7e^{-2}$ at 2.5 m for 20, 50, 100 and 200 kbps, respectively, which is a reduction by up to 93.2% from conventional ones. At 0 m, all algorithms achieved almost identical BER results since it has the best conditions for communication (except for the case of 200 kbps, where the proposed algorithm achieved BER reduction by 84.8%). The results show the trend that BER increases as the backscatter tag moves farther from the transmitter/receiver; we have the smallest BER at 0 m (at most, $8.7e^{-4}$) and higher BERs in farther positions from it. We also see that BERs at a higher bit rate are higher than those at a lower bit rate. The highest gain is achieved as reduction by 94.4% from the BER of the sliding method at 1 m.

**Figure 11:**
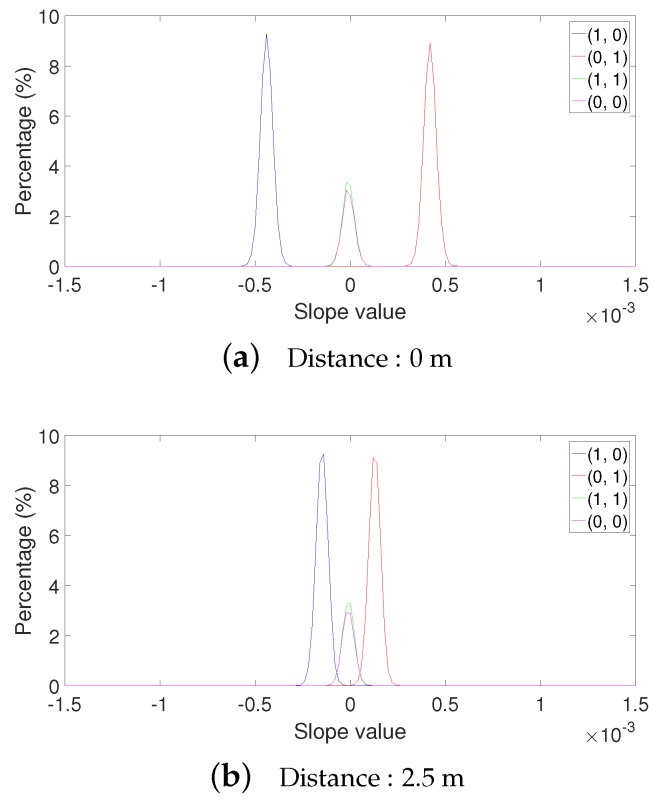
Distribution of slope values for different bit pairs at a bit rate of 100 kbps.

**Figure 12:**
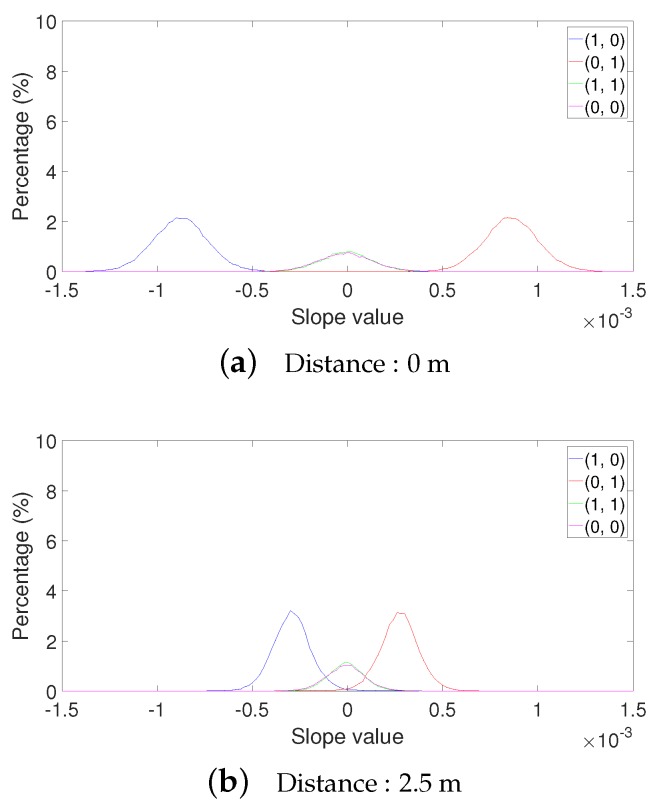
Distribution of slope values for different bit pairs at a bit rate of 200 kbps.

In Figure 11, the distributions of slopes for different bit pairs at 100 kbps are shown at 0 and 2.5 m. At 0 m, bit pairs have distinct slope distributions. All slope values of bit pair (1, 0) are negative, and those of bit pair (0, 1) are all positive. The slope values of bit pairs (1, 1) and (0, 0) are distributed around zero, but do not overlap with the distributions of bit pairs (1, 0) and (0, 1). Therefore, the proposed algorithm clearly differentiates bit pairs and achieves near-zero bit error at 0 m. At 2.5 m, however, the slope distributions of different bit pairs have much overlap with each other. Some slope values of bit pairs (1, 0) and (0, 1) appear positive and negative, respectively, thus leading to wrong decoding results and high BER at 2.5 m. When the bit rate is doubled as 200 kbps, the slope distributions of different bit pairs become less distinguishable between them, as shown in Figure 12. We also observed that the slope values of 200 kbps were more dispersed than those of 100 kbps. This is because signal fluctuation is less filtered out due to a smaller smoothing window size.

**Figure 13:**
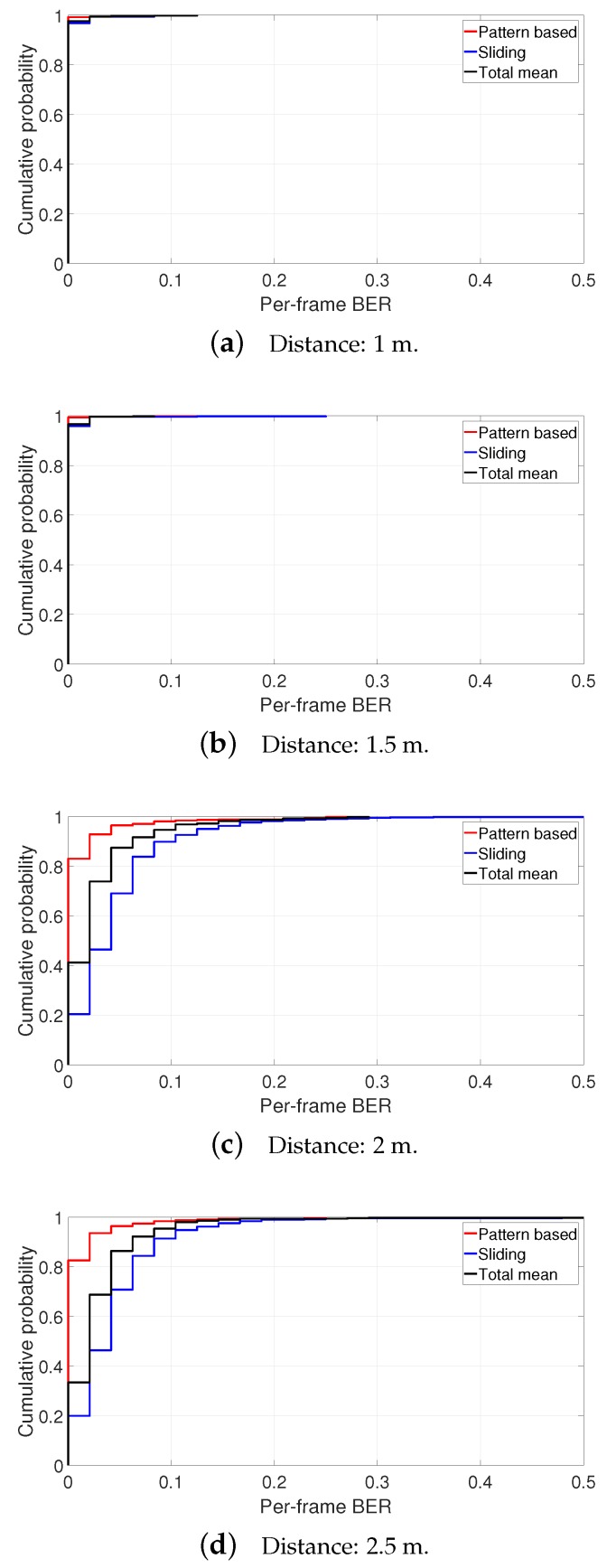
Cumulative probability distribution of per-frame BER for 100 kbps.

Figure 13 shows the cumulative distribution functions (CDFs) of per-frame BER (calculated as the ratio of errored bits to the total number of backscattered bits on a Wi-Fi frame) (If we assume that the tag’s packet is transmitted per Wi-Fi frame, this per-frame BER is readily converted into the packet error rate) for three different decoding methods with distances from 1 to 2.5 m (BER results at shorter distances are less problematic) at 100 kbps. As can be seen from the CDF results, transmission performance decreases as the distance of the backscatter tag becomes longer. As shown in the figure, the proposed algorithm enables the tag to transmit without any error for more than 80% of Wi-Fi frames at all distances. While threshold-based methods achieve similar results with the proposed algorithm at 1 and 1.5 m, they successfully decode data at most for or less than 40% of Wi-Fi frames at 2 and 2.5 m. Performance is degraded as the tag moves farther from the Wi-Fi transmitter and receiver since the signal’s traversing path becomes longer.

## 7. Conclusions

In this paper, we proposed a pattern-based decoding algorithm for backscatter communications using ambient Wi-Fi signals. The proposed algorithm identifies the pattern of received signal samples, and is thus more robust against signal fluctuation with no need for tricky threshold configuration. Moreover, the pattern for a pair of adjacent bits is exploited, and the algorithm decodes a bit pair at a time, which enables the algorithm to make a decision on a bit from two patterns, and makes the decision more reliable against fluctuation. The experimental study with real Wi-Fi traffic demonstrated that the proposed algorithm has noticeable performance enhancement over conventional threshold-based decoding methods. The proposed algorithm can be combined with or extended for other techniques of a longer communication range, higher-order modulation, etc. to achieve higher overall performance.

## Abbreviations

The following abbreviations are used in this manuscript:

AP	Access point
BER	Bit error rate
CDF	Cumulative distribution function
CSI	Channel state information
MAC	Medium access control
OFDM	Orthogonal frequency-division multiplexing
PAPR	Peak-to-average-power-ratio
PSK	Phase-shift keying
RF	Radio frequency
RSSI	Received signal strength indication
SIC	Successive interference cancellation
USRP	Universal Software Radio Peripheral

## Appendix A. Derivation of Equation (A6)

Applying Equation (4) to smoothed signal samples $\bar{y}\left[i\right]$ for $i\in [\tau_{n}-T/2,\tau_{n}+T/2-1]$, slope[*n*] of the samples is given as

(A1) $$\begin{array}{c} \mathrm{slope}\left[n\right]=\frac{\sum_{i=\tau_{n}-T/2}^{\tau_{n}+T/2-1}i\bar{y}\left[i\right]-T\bar{i}\bar{\bar{y}}}{\sum_{i=\tau_{n}-T/2}^{\tau_{n}+T/2-1}{\left(i-\bar{i}\right)}^{2}} \end{array}$$

where $\bar{i}$ and $\bar{\bar{y}}$ are the averages of *i* and $\bar{y}\left[i\right]$, respectively. Define $j:=i-\tau_{n}+\frac{T}{2}+1$ (we leave $\bar{y}\left[i\right]$ for simplicity of exposition). Then, we rewrite slope[*n*] as

(A2) $$\begin{array}{c} \mathrm{slope}[n]\\ =\frac{\sum_{j=1}^{T}\left(j+\tau_{n}-\frac{T}{2}-1\right)\bar{y}\left[i\right]-T\left(\bar{j}+\tau_{n}-\frac{T}{2}-1\right)\bar{\bar{y}}}{\sum_{j=1}^{T}{\left(j-\bar{j}\right)}^{2}}\\ =\frac{\sum_{j=1}^{T}j\bar{y}\left[i\right]-T\bar{j}\bar{\bar{y}}}{\sum_{j=1}^{T}{\left(j-\bar{j}\right)}^{2}}. \end{array}$$

where $\bar{j}$ and $\bar{\bar{y}}$ are obtained as

(A3) $$\begin{array}{c} \bar{j}=\frac{1}{T}\sum_{k=1}^{T}j=\frac{1}{T}\frac{T(T+1)}{2}=\frac{T+1}{2},\\ \bar{\bar{y}}=\frac{1}{T}\sum_{j=1}^{T}\bar{y}\left[i\right]. \end{array}$$

Therefore, the numerator of Equation (A2) is reduced to

(A4) $$\begin{array}{c} \sum_{j=1}^{T}j\bar{y}\left[i\right]-T\frac{T+1}{2}\frac{1}{T}\sum_{j=1}^{T}\bar{y}\left[i\right]=\sum_{j=1}^{T}\left(j-\frac{T+1}{2}\right)\bar{y}\left[i\right] \end{array}$$

and the denominator is obtained as

(A5) $$\begin{array}{c} \sum_{j=1}^{T}\left(j^{2}-2j\bar{j}+{\bar{j}}^{2}\right)\\ =\sum_{j=1}^{T}j^{2}-2\bar{j}\sum_{j=1}^{T}j+T{\bar{j}}^{2}\\ =\frac{T(T+1)(2T+1)}{6}-2\frac{T+1}{2}\frac{T(T+1)}{2}+\frac{T{\left(T+1\right)}^{2}}{4}\\ =\frac{T(T+1)}{12}\left(2\left(2T+1\right)-6\left(T+1\right)+3\left(T+1\right)\right)\\ =\frac{T(T+1)}{12}\left(T-1\right). \end{array}$$

Finally, applying Equations (A4) and (A5) into Equation (A2), and changing *j* back to *i*, we have

(A6) $$\begin{array}{ccc} \mathrm{slope}[n] & = & \;\frac{12}{T(T^{2}-1)}\sum_{j=1}^{T}\left(j-\frac{T+1}{2}\right)\bar{y}\left[i\right]\\ & = & \;\frac{12}{T(T^{2}-1)}\sum_{i=\tau_{n}-T/2}^{\tau_{n}+T/2-1}\left(i-\tau_{n}+\frac{1}{2}\right)\bar{y}\left[i\right]. \end{array}$$
